# Ictal EEG source imaging in presurgical evaluation: High agreement between analysis methods

**DOI:** 10.1016/j.seizure.2016.09.017

**Published:** 2016-12

**Authors:** Sándor Beniczky, Ivana Rosenzweig, Michael Scherg, Todor Jordanov, Benjamin Lanfer, Göran Lantz, Pål Gunnar Larsson

**Affiliations:** a Department of Clinical Neurophysiology, Danish Epilepsy Centre, Dianalund, Denmark; b Department of Clinical Neurophysiology, Aarhus University Hospital, Aarhus, Denmark; c Sleep and Brain Plasticity Centre, Department of Neuroimaging, IOPPN, King’s College and Imperial College, London, UK; d Research Department, BESA GmbH, Gräfelfing, Germany; e Clinical Neurophysiology Unit, Department of Clinical Sciences, Lund University, Lund, Sweden; f Electrical Geodesics, Inc., Eugene, OR, USA; g Clinical Neurophysiology Section, Department of Neurosurgery, Oslo University Hospital, Norway

**Keywords:** EEG, Epilepsy surgery, Inverse solution, Seizure, Source imaging

## Abstract

**Purpose:**

To determine the agreement between five different methods of ictal EEG source imaging, and to assess their accuracy in presurgical evaluation of patients with focal epilepsy. It was hypothesized that high agreement between methods was associated with higher localization-accuracy.

**Methods:**

EEGs were recorded with a 64-electrode array. Thirty-eight seizures from 22 patients were analyzed using five different methods phase mapping, dipole fitting, CLARA, cortical-CLARA and minimum norm. Localization accuracy was determined at sub-lobar level. Reference standard was the final decision of the multidisciplinary epilepsy surgery team, and, for the operated patients, outcome one year after surgery.

**Results:**

Agreement between all methods was obtained in 13 patients (59%) and between all but one methods in additional six patients (27%). There was a trend for minimum norm being less accurate than phase mapping, but none of the comparisons reached significance. Source imaging in cases with agreement between all methods was not more accurate than in the other cases. Ictal source imaging achieved an accuracy of 73% (for operated patients: 86%).

**Conclusion:**

There was good agreement between different methods of ictal source imaging. However, good inter-method agreement did not necessarily imply accurate source localization, since all methods faced the limitations of the inverse solution.

## Highlights

• There was good agreement between different methods of ictal EEG source imaging.
• Ictal source imaging achieved an accuracy of 73% (for operated patients: 86%).
• Agreement between all methods did not necessarily imply accuracy of localization.

## 1 Introduction

There is compelling evidence for the role of electric source imaging (ESI) in the localization of interictal epileptiform discharges [1], [2], [3], [4], [5]. However, the irritative zone generating the interictal EEG discharges might not necessarily coincide with the seizure-onset zone [6]. Ictal source imaging faces additional technical challenges (artifacts occurring during seizure, absence of ictal EEG correlate in scalp recordings, propagation of ictal activity), and it has received less attention compared to interictal analysis [5].

Several methods of ictal source imaging have been previously described and validated in clinical practice [7], [8], [9], [10], [11], [12], [13]. However, it is not known to what extent the different methods lead to the same source location, and which is the best approach for localizing ictal sources. It was hypothesized that concordance between different methods/inverse solution was associated with a higher localization-accuracy [14].

The objectives of this study were: to investigate the agreement between different analysis strategies of ictal source imaging, to assess their accuracy in the presurgical evaluation of patients with epilepsy, and to test the hypothesis that higher inter-method agreement was associated with higher localization-accuracy.

## 2 Methods

### 2.1 Patients and recordings

Thirty-eight seizures from 22 consecutive patients (10 females) who met the inclusion criteria, were analyzed. The age of the patients was between 17 and 49 years (mean: 33.8 years). The mean duration of epilepsy, from the onset to the Long Term Monitoring was 17 years (median: 12.5, range: 2–48 years). Inclusion criteria were: patients who undergone long-term video-EEG monitoring for presurgical evaluation, who had had at least one seizure recorded, and for whom the multidisciplinary epilepsy surgery team was able to decide on the localization of the epileptogenic zone. Exclusion criteria was the absence of identifiable ictal EEG activity.

Patients gave their informed consent prior to the admission to the epilepsy monitoring unit (EMU). EEGs were recorded using 64 scalp electrodes according to the 10–10 setting.

Seventeen patients (77%) had epileptogenic lesion on the MRI. Supporting document 1 shows demographic and clinical information (including neuroimaging and electrophysiology) for all patients.

Supporting document 1 Clinical data and reference standards for all patients.

### 2.2 Ictal source imaging

Anonymized ictal EEG recordings were retrospectively analyzed, blinded to all clinical data, using BESA Research 6.1 software. Five different source analysis methods were applied: phase-mapping (PM), dipole fitting, CLARA, cortical-CLARA and minimum norm estimation (MN). The analysis methods are described in detail elsewhere [12], [13]. Briefly:

#### 2.2.1 Phase mapping

The first detectable oscillatory pattern at seizure-onset was marked and the spectral peak was determined using FFT. By combining the real and imaginary peak FFT coefficients at different phase angles, phase maps were calculated, i.e., voltage maps at various relative latencies by transforming phase into time [13], [15].

#### 2.2.2 Averaging of seizure onset waveforms and source imaging

The alternative approach to PM was based on averaging the ictal onset waveforms [12]. The averaged signals were analyzed using various inverse methods: discrete multiple dipole fitting to analyze onset and peak [16], [17], a distributed source model in the brain volume, i.e., classical LORETA analysis recursively applied (CLARA), a similar distributed source model, but constrained to the cortex (cortical CLARA), and a cortex-constrained minimum norm estimation [18], [19].

Iterative application of LORETA in the brain volume as used in CLARA [20], [21] is a well-known and widely used method [22], [23]. Here, two iterations were performed. The initial image was regularized using a SVD cutoff of 0.005%; the two iterations were regularized with a cutoff of 0.01%.

Cortical CLARA was applied as a modification of the volume CLARA algorithm by constraining the source space to the cortical surface. For this, a graph Laplacian operator [24] was used that smooths along the cortical surface in contrast to the volume CLARA where the Laplacian smooths in all three dimensions [25]. The initial cortical CLARA image and the 10 following iterations used a SVD cutoff of 0.005%.

Thus, dipole fitting and CLARA provided equivalent centers of activation in the brain volume, whereas cortical CLARA and MN provided equivalent centers of activation along the cortical folds.

The cortex-constrained minimum norm was applied on the averaged data with depth and spatio-temporal weighting based on the signal subspace correlation measure [26]. Noise was estimated from the baseline interval. For each channel, separate noise weights were used for the diagonal noise covariance matrix.

#### 2.2.3 Head model

The new standard head model of BESA Research 6.1 for adults (age 20–24) was used [27]. This is based on a head template created by non-linear morphing and averaging of 10 adult heads into one standard head with the goal to render the cortical folds optimally. Currently, this standard template is the only one having sufficiently good rendering of all tissues needed for the computation of the forward, finite-elements model (FEM) in BESA MRI [28], [29]. The full set of standard 10–10 electrodes was warped onto the head template according to the rules of the 10–10 system how to place electrodes relative to the landmarks, i.e., nasion, inion, and pre-auricular points. These landmarks could be identified on the reconstructed standard head surface. Thus, standard electrode coordinates and FEM lead fields vectors were available to compute the forward model for the 64 electrodes used in this study.

### 2.3 Reference standard (“gold standard”)

We compared the source images with two sets of reference standards. For all patients, source images were compared with the final decision of the multidisciplinary epilepsy surgery team. In addition, for the 20 patients who underwent respective epilepsy surgery, we also compared the centers of the source images with the resected areas and the surgical outcome one year after the operation [30]. Patients were considered seizure-free if they were in Engel class I.

### 2.4 Evaluation of the source models

The source images were evaluated by one of the authors (IR) who was blinded both for the clinical and for the raw-EEG data. Center source locations were scored at sub-lobar level [31]. In temporal lobe cases, we considered a source as mesial temporal if it localized to the mesial, basal or antero-polar part of the temporal lobe; other temporal localizations were scored as lateral-neocortical in concordance with previous studies, using simultaneous scalp and intracranial recordings [7], [32], [33], [34], [35].

The scored sub-lobar source locations were compared with the reference standard, and classified as concordant, partially concordant or discordant. A full match at sub-lobar level between the source locations and the gold standard was considered concordant. When the source images involved several sub-lobar structures, including the one in the reference standard, or, in patients with several seizures when at least one seizure was concordant and the other(s) were not, source location was considered partially concordant. All other cases were considered discordant.

Nine patients had two or more seizures with identifiable ictal EEG correlate. We analyzed each seizure separately in these patients; when all seizures in a patient were concordant with the reference standard, the patient was considered “concordant”; when only a part of the seizures were concordant with the reference standard, the patient was scored as “partially concordant”; when all seizures were discordant with the reference standard, the patient was considered “discordant”.

We compared the incidence of concordant cases among the five methods using Fisher’s exact test [36].

## 3 Results

**Fig. 1:**
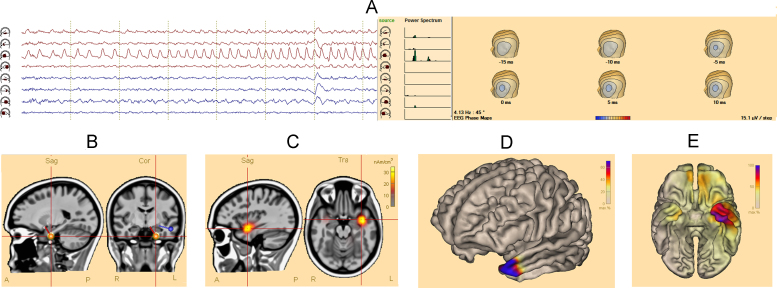
Ictal source imaging in a patient with left temporal focus. (A) Phase mapping: the source-channel corresponding to the lateral anterior part of the left temporal lobe shows the build-up of the ictal activity. The power-spectrum demonstrates a peak at 4.1 Hz, predominating at the lateral anterior part of the left temporal lobe; additional activity is seen at the basal part of the left temporal lobe. Phase-maps show a topography that is consistent with the left anterior temporal lobe. (B) Spatiotemporal dipole model: the red dipole corresponds to the onset phase of the averaged ictal waveform. It is located at the anterior-inferior part of the left temporal lobe. The blue dipole corresponds to the propagation phase (peak of the averaged discharge), and it is localized more laterally compared to the onset. (C) CLARA: the source-model is localized in the anterior-superior part of the left temporal lobe. (D) Cortical-CLARA: the distributed source model localizes to the left temporal pole. (E) Minimum norm: the distributed source model is more widespread, however, still localized to the antero-polar region of the left temporal lobe.

**Fig. 2:**
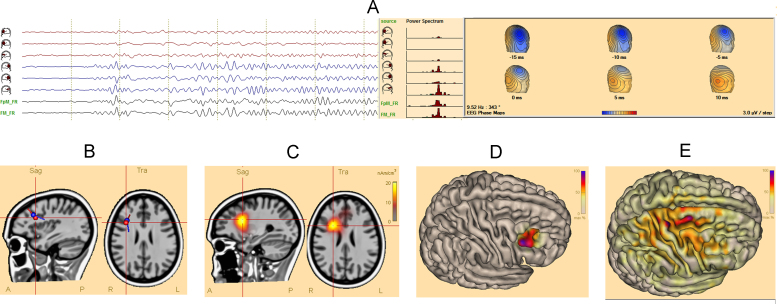
Ictal source imaging of a patient with right frontal focus. (A) Phase mapping: the source-channels corresponding to the right-frontal and mid-frontal regions show the build-up of the ictal activity. The power-spectrum demonstrates a peak at 9.5 Hz in these channels. Phase-maps show a distribution corresponding to the lateral part of the right frontal lobe. (B) Spatiotemporal dipole model: the red (onset) and blue (propagation) dipoles are localized in the same region of the right frontal lobe. Their orientation is different, suggesting propagation to the opposite wall of the sulcus. (C–E) Distributed source models are localized to the lateral part of the right frontal lobe ((C) CLARA; (D) cortical-CLARA; (E) minimum norm).

Fig. 1, Fig. 2 show source imaging results in patients with a temporal and a frontal focus. Supporting document 1 contains clinical data and reference standards for all patients. Fourteen patients had temporal foci, and 8 patients had extra-temporal foci (frontal: 5, parietal: 2, occipital: 1). Supporting document 2 shows source imaging in a patient with deep focus (periventricular heterotopia).

Supporting document 2

In 13 patients (59%) all methods of source imaging agreed on localization at sub-lobar level. In additional six patients (27%) there was agreement among all-but-one method. In three patients there was agreement between 3 methods.

The accuracy of the various methods is summarized in Table 1, Table 2. Source models yielded accurate solutions, concordant at sub-lobar level with the reference standard in 45–72% of the patients. This increased to 68–77% when including patients with partially concordant cases.

Twenty patients were operated on, and 14 became seizure-free (70%). In this subgroup of 14 patients, considering the location of the resection as reference standard, the accuracy of the source models was between 57–71%. When including patients with partially concordant source images, accuracy increased to 71–93%.

Although there was a trend for less accurate localization with MN, none of the comparisons reached level of significance. Similar results were obtained, when comparing ictal source imaging with the intracranial recordings (Supporting document 3).

Supporting document 3

In three out of the six operated patients who did not become seizure-free, ictal source imaging was discordant with the site of the resection (50%). The proportion of ictal source imaging results discordant with the site of resection was lower among operated patients who became seizure free (two out of 14 patients; 14%).

Ten out of the 13 patients with agreement between all methods had accurate source localizations (77%). This figure was not significantly higher compared to the other patients. Nine of the 13 patients with agreement among all methods were operated and became seizure-free. However, in one of these patients all source models localized outside the resected area; in all others source models coincided with the reference standard.

Five patients did not have epileptogenic lesion on the MRI. Ictal source imaging was concordant with the reference standard in four of these patients, and discordant in one patient. The non-lesional patient with discordance between the resected site and the ictal source imaging did not become seizure-free (Engel IV).

In six out of the eight patients with extratemporal foci (75%), ictal source imaging indicated locations that were concordant with the reference standard, and all but one of the six patients became seizure-free after operation. This was similar to the results in the sub-group of patients with temporal foci, where 10 out of the 14 patients had correct localization using ictal source imaging (71%).

In patients with more than one analyzed seizure, we investigated whether the seizure-by-seizure analysis of the ictal location was different in successive seizures. In five patients, all seizures had the ictal source model in the same sub-lobar area; four of these five patients had source locations concordant with the reference standard, and one patient had partially concordant source locations. Four patients had ictal sources in different locations, for the different seizures; none of these patients were concordant with the reference standard, three patients were partially concordant and one patient was discordant. Thus, the incidence of concordant cases was significantly higher among the patients in whom all seizures had the same ictal source (p = 0.046).

## 4 Discussion

There is a wide variety of available methods and inverse solutions for source imaging of epileptiform EEG activity. Due to the underdetermined nature of the inverse problem, each method operates with specific additional constraints in order to localize the source. But what is the best approach? Does agreement between several methods or different inverse solutions imply an accurate localization?

The inter-observer variability in clinical EEG-reading has been addressed in many studies [37], [38]. However, the agreement between different source imaging methods has received little attention so far. Averaged interictal epileptiform discharges from two patients have been analyzed independently by different groups, who applied different source localization strategies [14]. Most of the methods led to correct localizations of the interictal epileptiform discharges.

We have investigated the inter-method agreement for ictal source imaging, using different analysis strategies and inverse solutions, based on time-frequency methods (phase mapping), spatiotemporal dipole model and various distributed source models (CLARA, cortical-CLARA, MN). Our results suggest that there is a good agreement between various methods of ictal source imaging. In spite of the different type of constrains/inverse solutions, in 86% of cases there was agreement at sub-lobar level between at least four of the five applied methods, and in 59% of cases all methods were in agreement.

Full agreement among all applied methods does not guarantee an accurate source localization. This suggests that the major limitation of ictal source imaging relies in the underdetermined nature of the inverse solution, which cannot be circumvented by applying several methods.

Although MN seemed to have lower accuracy than the other methods, none of the comparisons reached level of significance, so we cannot point out any of the methods as superior to the other ones. The ictal source imaging in our study reached an accuracy of 73% (and for the seizure-free, operated cases: 86%). This is comparable with other functional imaging methods [4].

Although ictal source imaging in patients with extratemporal foci, faces additional technical challenges, in our series, we found that accuracy of ictal source imaging in this sub-group of patients was similar to those with temporal foci. Visualizing the ictal signals in source space has improved the identification and analysis of the ictal signals (Fig. 2A).

Since the study was retrospective, the results of the ictal source imaging did not influence the clinical decision making, and hence, clinical utility in these patients could not have been determined. Nevertheless, the analysis was done blinded to all other clinical data, thus the high accuracy (73–86%) was not influenced by information from other modalities. In the sub-group of non-lesional patients, accuracy (80%) was similar to the patients with epileptogenic lesion on the MRI.

In this study we used the same conductivity values for source imaging in all patients. An in-vitro study using freshly excised cerebral cortex in epilepsy surgery patients, suggested that electrical conductivity varies as a consequence of clinical variables, such as underlying pathology and seizure duration [39]. Better understanding of how disease affects cortical electrical conductivity and ways to measure it non-invasively (for example using diffusion tensor imaging), could increase the accuracy of the inverse solutions [39].

Ictal source imaging was able to localize correctly, at sub-lobar level, even deep foci (Supporting document 2 shows MRI and source imaging in a patient with periventricular heterotopia).

Our results in patients with multiple analyzed seizures suggested that patients in whom ictal sources from all seizures localized to the same sub-lobar region, were more often concordant with the reference standard. However, the sample size was relatively small (nine patients with multiple seizures) and further studies are necessary to elucidate the impact of inter-seizure agreement.

In conclusion, our results support the clinical reliability of ictal source imaging methods and advocate for their implementation in the presurgical evaluation of patients with intractable focal epilepsy.

## Conflict of interest statement

Author MS is a shareholder and employee of BESA GmbH, a company developing and providing software tools for EEG and source analysis. Authors TJ and BL are employees of BESA GmbH. Author GL is employee of Electrical Geodesics, Inc. The other authors do not have any conflict of interest to disclose.

## Figures and Tables

**Table 1:** Number (%) of concordant patients.

	Phase maps	Dipole	CLARA	Cortical CLARA	Minimum norm
All patients (n = 22)	16 (73%)	13 (59%)	13 (59%)	13 (59%)	10 (46%)
Seizure-free patient (n = 14)	12 (86%)	10 (71%)	10 (71%)	9 (64%)	8 (57%)

**Table 2:** Number (%) of concordant and partially concordant patients.

	Phase maps	Dipole	CLARA	Cortical CLARA	Minimum norm
All patients (n = 22)	16 (73%)	17(77%)	16 (72%)	17 (77%)	15 (68%)
Seizure-free patient (n = 14)	12 (86%)	10 (71%)	12 (86%)	13 (93%)	12 (86%)
