# Supplementary material for: Ictal EEG source imaging in presurgical evaluation: High agreement between analysis methods
Source: Seizure. 2016 Dec;43:1–5. doi: 10.1016/j.seizure.2016.09.017 (PMC5176190; doi:10.1016/j.seizure.2016.09.017)
Supplement: Supporting document 1 — Clinical data and reference standards for all patients. [file mmc1.pdf]

Pt.	Age	G	Conclusion of the MDT on the location	PM	Dipole	CLARA	C-CLARA	MN	MA	Engel class	MRI	PET	SPECT	Intracranial recordings
1	44	f	Left mesial temporal	C	C	C	C	C	5	Engel IB	Left hippocampal sclerosis	FMZ:left temp	No spect	left temp
2	36	m	Left frontal lateral (rostral to Broca)	D	D	D	D	D	4	Engel IV	High intensity areas fronto-temporal L>>R (encephalomalacy	No PET	No spect	Left frontal lateral
3	34	f	Left frontal (at the heterotopia)	C	C	C	C	C	5	Engel IA	Left ucleus caudatus heterotopia	Left Temp	Left.temp	Left- at the heterotopia
4	23	m	Left temporal operculum	C	P	P	P	P	4	Engel IB	Left temp (DNET)	Rigth temp-occ	Right temp	
5	25	m	Right frontal - cranial-mesial	C	P	P	P	P	3	Engel IA	Right front (tumor)			
6	48	m	Right temp lateral / basal	C	C	C	C	P	4	Engel IA	Normal (Minimally enlarged right temporal horn)	Righth temp (FMZ+FDG)	No SPECT	
7	44	f	Left temp antero-mesial	C	C	C	C	C	5	Engel IA	Multiple small lesions in right superior temporal gyrus	Right insula	Left temp	Left temporal
8	22	m	Left temp (amygdala)	C	C	C	C	C	5	Engel IA	Left temp hippocampal sclerosis			Left temporal
9	21	f	Right temp superior	D	P	D	D	D	4		Right temporal tumor (astrocythoma)			
10	46	m	Left temporal antero-mesial	C	C	C	C	C	5	Engel IA	Left middle front gyrus increased signal (neurovascular)	Left temporal		Left temp. antero-mesial
11	49	f	Left temporal mesial-basal	D	D	D	P	D	4	Engel IA	Left hippocampal sclerosis	Left temp		Left temporal
12	46	f	Right parietal lateral	C	C	C	C	C	5	Engel IA	Right postcentral sulcus - Focal Cortical Dysplasia			Right parietal
13	43	f	Left temp mesial	D	D	D	D	D	5	Engel III	Tumor in left amygdala + pes hippocampi	Left temp		
14	36	f	Right temp mesial	C	C	C	C	C	5	Engel IVB	Right temporo-occipital Focal Cortical Dysplasia	Right temp	Right temp	Right temp
15	18	f	Right temp basal-polar	C	C	C	C	C	5	Engel IA	Normal	Rigth temp		Right temp
16	37	m	Right temp basal	C	C	C	C	C	5	Engel IV	Right fronto-temporo-basal (trauma)	Right front+temp	Right temp	Right temp
17	24	m	Left frontal - g.front. Med.	C	C	C	P	P	3	Engel IB	Polymicrogyria: Left med . Frontal	Normal		Left gy. front med
18	40	m	Right temporal lateral (middle)	C	P	P	C	P	3		Normal	Normal		Right temproal lateral
19	43	m	Right frontal operculum - F2	C	C	C	C	C	5	Engel IA	Normal (Hippocampus malrotation)	Normal	Right temp	Right temp-occ
20	17	m	R.parietal -primary sensory ctx -hand	D	D	D	D	D	5	Engel IA	Multiple Tuber right front , bilat	AMT: r.front+r.temp+l.par	No spect	R.parietal
21	24	f	Left temporal	D	D	D	D	D	5	Engel IV	Normal (Left frontal-minor unspecific white matter)	Left temp	No SPECT	Left temporal
22	24	m	Right occipital	C	C	C	C	D	4	Engel IV	Right occ encephalomalacia (old trauma)	Unknown	Right occ (ictal)	Right occipital

Legend

Pt.= consecutive patient number
G=gender
Age - in years
f=female
m=male
MDT=multidisciplinary team
PM= Phase mapping

C-CLARA=cortical-CLARA
MN=Minimum norm estimation
MA=model-agreement: number of methods with agreement at sublobar level
C=concordant with the reference standard, at sub-lobar level
P=partially concordant with the reference standard, at sub-lobar level
D=discordant with the reference standard
