# Supplementary material for: Ictal EEG source imaging in presurgical evaluation: High agreement between analysis methods
Source: Seizure. 2016 Dec;43:1–5. doi: 10.1016/j.seizure.2016.09.017 (PMC5176190; doi:10.1016/j.seizure.2016.09.017)

Supporting document 2

MRI and source imaging of the patient with periventricular heterotopia

A.

MRI showing periventricular heterotopia (red arrow) posterior to the left caudate nucleus (axial T1 image).

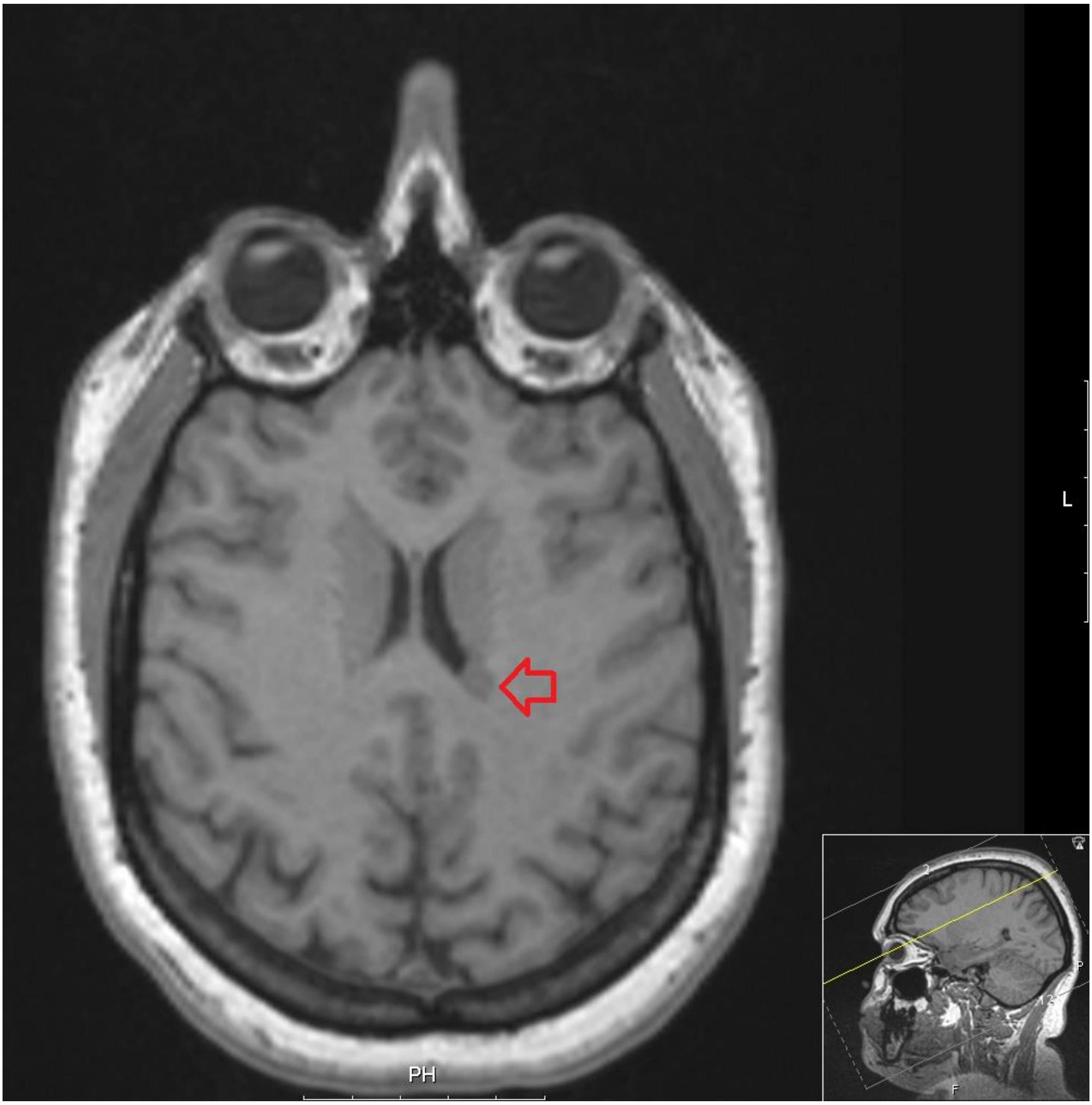

B.

The ictal signals visualized in source montage and the corresponding phase-maps. Observe that the rhythmic ictal signal is shown to be generated mainly in the source space corresponding to the deep structures (tangential orientation) in the left frontal lobe (green box).

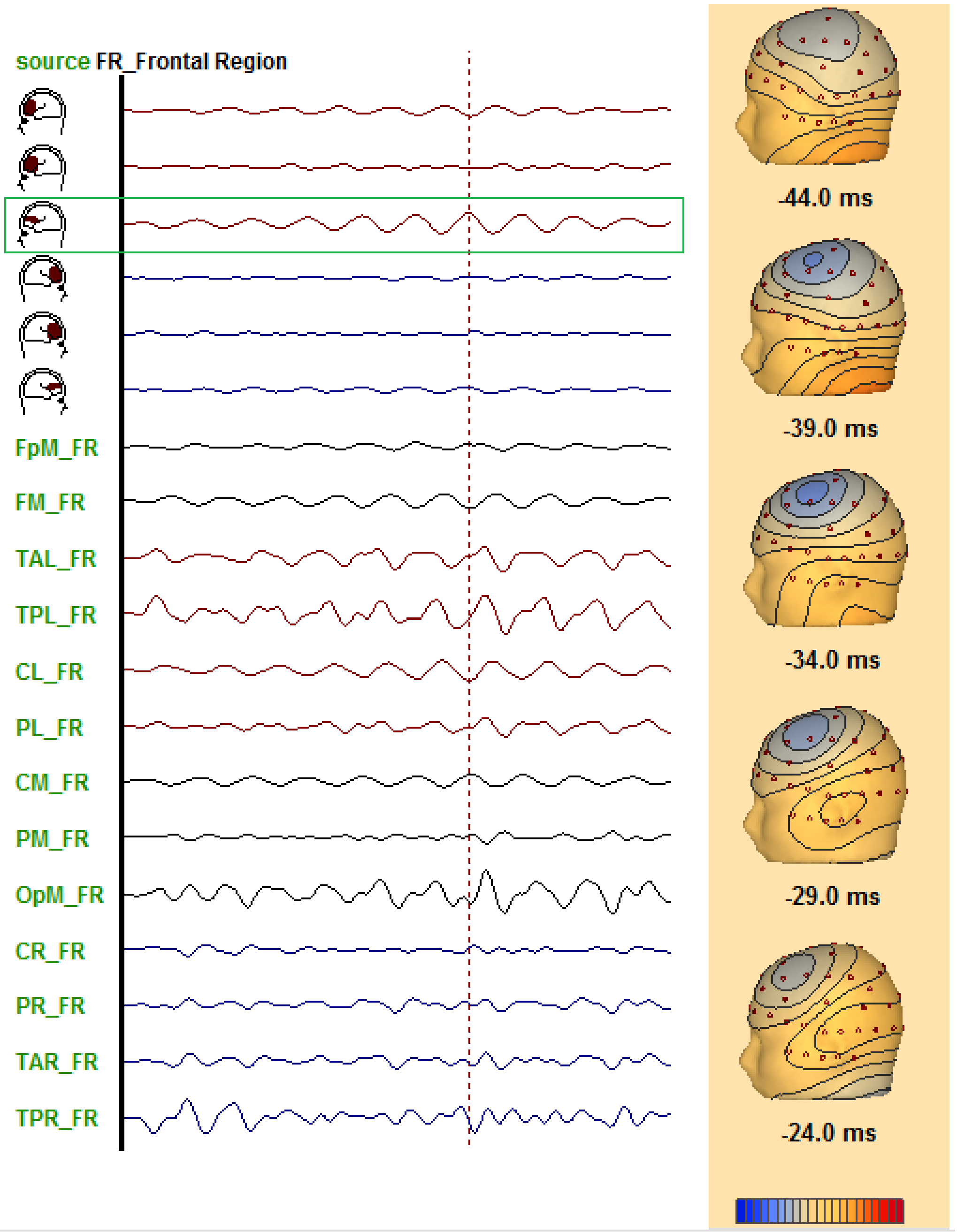

C.

Dipole and CLARA source models showing the ictal source corresponding to the location of the heterotopia

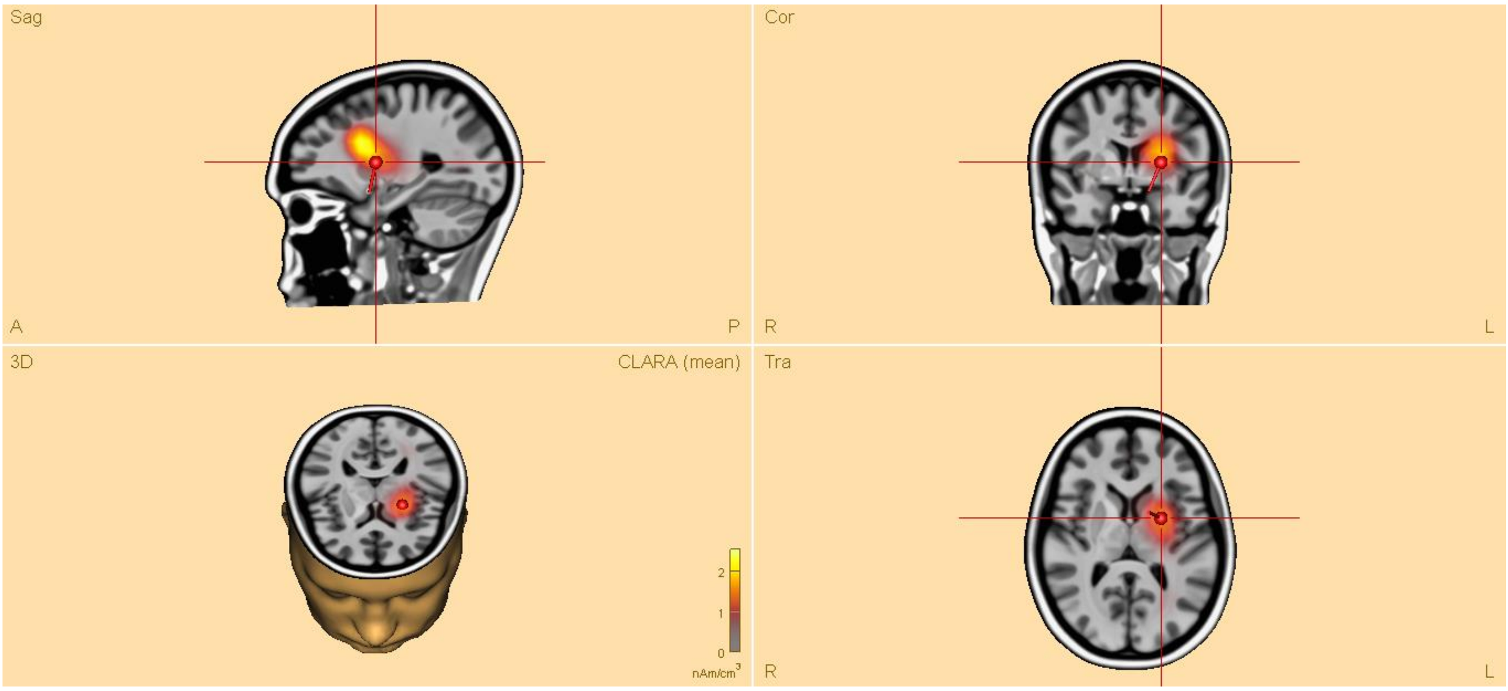

D.

Minimum norm (left) and Cortical CLARA (right) are visualized on the inflated cortex, in order to display deeper located cortical sources. The models co-localize at sub-lobar level with the heterotopia, in the left frontal lobe.

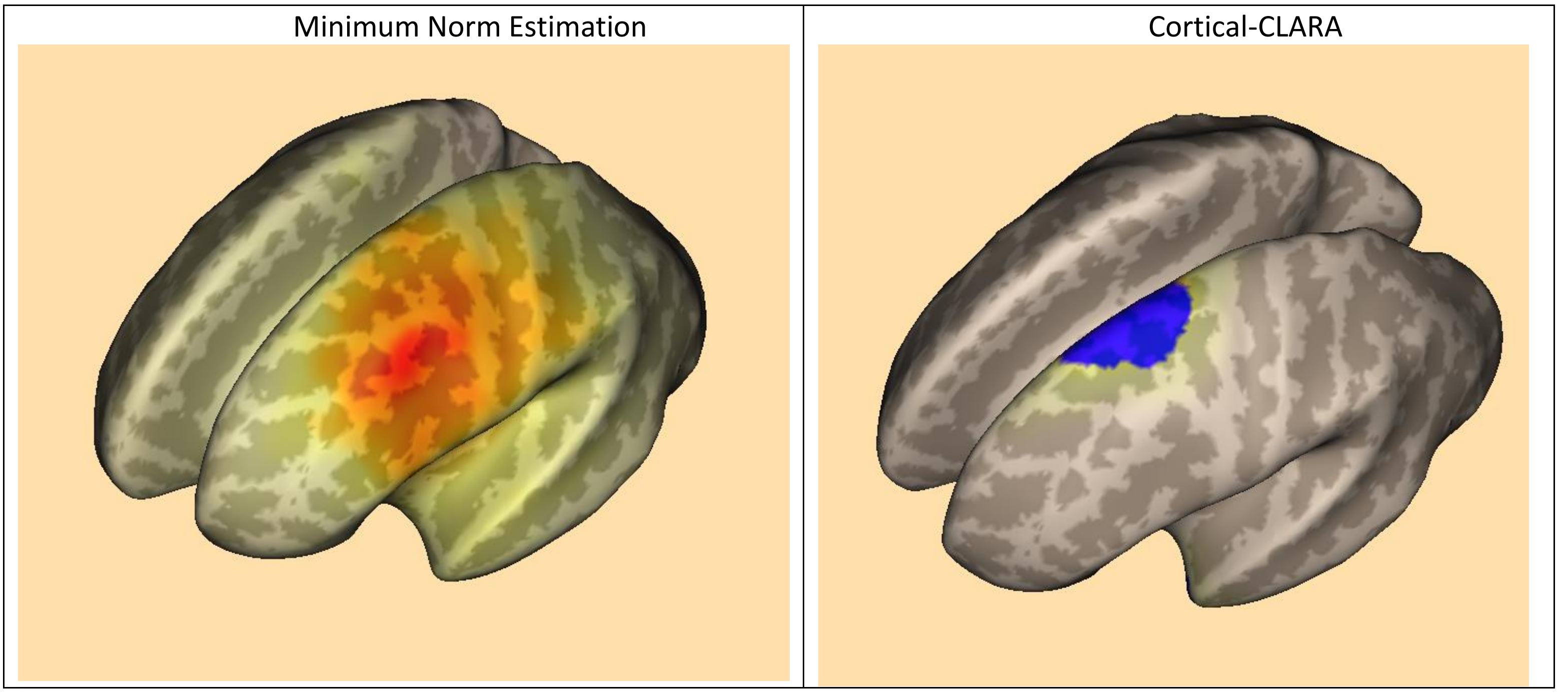

Supplement: Supporting document 2 [file mmc2.pdf]
