# Supplementary material for: Ictal EEG source imaging in presurgical evaluation: High agreement between analysis methods
Source: Seizure. 2016 Dec;43:1–5. doi: 10.1016/j.seizure.2016.09.017 (PMC5176190; doi:10.1016/j.seizure.2016.09.017)
Supplement: Supporting document 3 [file mmc3.pdf]

Supporting document 3

Number (%) of patients where ictal source imaging was concordant with the intracranial recordings. Seventeen patients had intracranial recordings.

	Phase maps	Dipole	CLARA	Cortical CLARA	Minimum Norm
Concordant	12 (71%)	11 (65%)	11 (65%)	11 (65%)	9 (53%)
Concordant and partially concordant	12 (71%)	12 (71%)	12 (71%)	13 (76%)	11 (65%)
